# An International Study on the Determinants of Poor Sleep Amongst 15,000 Users of Connected Devices

**DOI:** 10.2196/jmir.7930

**Published:** 2017-10-23

**Authors:** Guy Fagherazzi, Douae El Fatouhi, Alice Bellicha, Amin El Gareh, Aurélie Affret, Courtney Dow, Lidia Delrieu, Matthieu Vegreville, Alexis Normand, Jean-Michel Oppert, Gianluca Severi

**Affiliations:** ^1^ Centre de Recherche en Epidémiologie et Santé des Populations U1018 Inserm Villejuif France; ^2^ Bioingénierie, Tissus et Neuroplasticité Université Paris-Est Créteil Creteil France; ^3^ Leon Berard Cancer Center Lyon France; ^4^ Nokia Issy-les-Moulineaux France; ^5^ Institute of Cardiometabolism and Nutrition Department of Nutrition, Pitie-Salpetriere University Hospital, Assistance Publique-Hôpitaux de Paris Paris France; ^6^ University Pierre et Marie Curie-Paris Paris France

**Keywords:** connected devices, sleep, Withings, Nokia, determinants, Internet of Things, epidemiology, wearables, lifestyle, blood pressure, steps, heart rate, weight

## Abstract

**Background:**

Sleep is a modifiable lifestyle factor that can be a target for efficient intervention studies to improve the quality of life and decrease the risk or burden of some chronic conditions. Knowing the profiles of individuals with poor sleep patterns is therefore a prerequisite. Wearable devices have recently opened new areas in medical research as potential efficient tools to measure lifestyle factors such as sleep quantity and quality.

**Objectives:**

The goal of our research is to identify the determinants of poor sleep based on data from a large population of users of connected devices.

**Methods:**

We analyzed data from 15,839 individuals (13,658 males and 2181 females) considered highly connected customers having purchased and used at least 3 connected devices from the consumer electronics company Withings (now Nokia). Total and deep sleep durations as well as the ratio of deep/total sleep as a proxy of sleep quality were analyzed in association with available data on age, sex, weight, heart rate, steps, and diastolic and systolic blood pressures.

**Results:**

With respect to the deep/total sleep duration ratio used as a proxy of sleep quality, we have observed that those at risk of having a poor ratio (≤0.40) were more frequently males (odds ratio [OR]_female vs male_=0.45, 95% CI 0.38-0.54), younger individuals (OR_>60 years vs 18-30 years_=0.47, 95% CI 0.35-0.63), and those with elevated heart rate (OR_>78 bpm vs ≤61 bpm_=1.18, 95% CI 1.04-1.34) and high systolic blood pressure (OR_>133 mm Hg vs ≤116 mm Hg_=1.22, 95% CI 1.04-1.43). A direct association with weight was observed for total sleep duration exclusively.

**Conclusions:**

Wearables can provide useful information to target individuals at risk of poor sleep. Future alert or mobile phone notification systems based on poor sleep determinants measured with wearables could be tested in intervention studies to evaluate the benefits.

## Introduction

Extreme sleep duration has been increasingly recognized as a behavioral factor of interest, along with diet, physical activity, and being overweight, involved in the pathogenesis of various chronic noncommunicable diseases such as cancer [1,2], type 2 diabetes [3-5], and hypertension [6]. Sleep has been shown to be influenced by age, sex, obesity [7], hypertension [6], physical activity [8], alcohol consumption [9], and anxiety [10,11]. Poor sleep has been shown to have similar effects on health as major sleep disorders but is often neglected in primary and tertiary prevention programs [12].

As a lifestyle-related and modifiable factor, sleep can be a target for efficient intervention studies to improve the quality of life [13] and health of people [14,15]. Therefore, knowing the profiles of individuals with poor sleep quantity or quality is a prerequisite for an optimal identification of key populations of interest. However, in large cohort studies, sleep is often evaluated through self-report as the number of hours that participants typically sleep per night [16]; little is known about the duration of deep sleep or the quality of sleep.

As the information and communication technology market has exploded in recent years, more and more wearable activity trackers provide information about sleep but with limited evidence of their accuracy [17,18]. Information about sleep is estimated thanks to proprietary algorithms from data generated from in-built accelerometers to determine sleep parameters. It has been shown that some fitness trackers overestimate total sleep time when evaluated by polysomnography, in particular on nights with more disrupted sleep [19], and that they tend to underestimate sleep disruptions and overestimate total sleep times and sleep efficiency in normal subjects [20-22]. Nevertheless, previous work has suggested that trackers can be a low-cost and wide-availability alternative to standard activity monitoring of daily sleep-wake rhythms over several days, especially in large population or cohort studies.

Therefore, based on data from an international sample of sleep information from more than 15,000 customers of the consumer electronics company Withings (now Nokia), we have evaluated several determinants of poor total and deep sleep quantity and determined a ratio of deep/total sleep duration that indicates poor sleep.

## Methods

### Study Sample and Available Data

The population was composed of a sample of 16,441 Withings highly-connected customers—those who had purchased at least 3 Withings devices (a Pulse activity tracker [23,24], a Body weighing scale [25], and a BP-800 blood pressure monitor [26]) and had some data on sleep [27] available between July 1, 2013, and April 1, 2016. Individuals were excluded from the analysis if they did not have 7 consecutive days of complete sleep data. We excluded a few individuals with missing or unlikely information on weight, heart rate, steps [28], and diastolic or systolic blood pressure prior to the selected week of sleep evaluation. After these few exclusions, the final study sample was composed of 15,839 individuals (13,658 males and 2181 females).

### Assessment of Sleep

We selected the first 7 consecutive days with complete data on sleep available and computed the average duration of total and deep sleep per night. Sleep duration is defined thanks to a proprietary algorithm using data provided at a minute-level from both the accelerometer and the temperature sensor present inside the wearable (the body temperature drops during sleep). Deep sleep is further defined based on the information provided by the accelerometer in the device and corresponds to a period with a lower motion quantity. The ratio of deep/total sleep duration was also derived from average duration of total and deep sleep durations. Binary variables were then computed to categorize individuals’ sleep as short or adequate. We used the common threshold of 6 hours per night to define a short total sleep duration [29]. Short deep sleep duration was defined as lass than 3 hours per night, which is the closest integer to the 1st quartile of the deep sleep duration distribution in our study. Similarly, a ratio of deep/total sleep duration indicating poor sleep was defined as below 0.40, which corresponds to the 1st quartile of the distribution.

In the dataset, information on age (18 to 30 years, 31 to 40 years, 41 to 50 years, 51 to 60 years, and >60 years), sex, weight (kg), heart rate (bpm), steps (n/day), diastolic blood pressure (mm Hg), systolic blood pressure (mm Hg) was available. As our exposure, we used the average value of all the data available in the month prior to the week of sleep considered.

### Statistical Analysis

Characteristics of the study population were described according to categories of total sleep, deep sleep, and ratio of deep/total sleep duration and are displayed in Table 1. Logistic regression models were computed and odds ratios (OR) and their 95% confidence intervals (CI) were estimated. Multivariate models were adjusted for age, sex, weight, heart rate, steps, diastolic blood pressure, and systolic blood pressure (Table 2). SAS 9.4 (SAS Institute) software was used. Statistical tests were 2-sided, and *P* values were considered significant if *P*<.05.

## Results

### Characteristics of the Study Population

As described in Table 1, individuals with a low total sleep duration (≤6 hours) were more frequently males aged between 31 and 60 years with greater weight, heart rate, and number of steps than those with a high sleep duration, whereas average blood pressure was rather similar between the 2 groups. Concerning deep sleep duration, those with a duration ≤3 hours were also more frequently males but more frequently aged 51 years or more. They were also characterized by a greater weight, heart rate, number of steps per day, and blood pressure compared to those with a high deep sleep duration. Finally, individuals with a low deep/total sleep duration ratio were more frequently males aged between 31 and 50 years and had a greater weight, heart rate, and blood pressure and a lower number of steps.

**Table 1:** Characteristics of the study population (N=15,839).

Factors		Total sleep duration		Deep sleep duration		Deep/total ratio	
		≤6h n=4169	>6h n=11670	≤3h n=5845	>3h n=9994	≤0.40 n=3309	>0.40 n=12530
**Age, years (%)**							
	18-30	1.66	2.22^a^	1.93	2.31^a^	3.08	1.80^a^
	31-40	13.60	12.28	11.40	14.73	14.90	12.03
	41-50	30.56	29.53	29.12	30.97	31.22	29.43
	51-60	32.14	31.25	31.90	30.78	29.65	31.97
	>60	22.04	24.72	25.66	21.21	21.15	24.77
Sex (% female), mean (SD)		10.75	14.85^a^	8.96	16.58^a^	7.89	15.32^a^
Weight (kg), mean (SD)		89.26 (19.35)	87.33 (18.19)	89.07 (18.93)	87.12 (18.23)	89.41 (19.01)	87.42 (18.37)
Heart rate (bpm), mean (SD)		71.32 (13.97)	70.26^b^ (13.83)	71.27 (14.23)	70.12 (13.66)^b^	71.26 (14.29)	70.34 (13.76)^b^
Steps (n/day), mean (SD)		7237.32 (3333.24)	7159.29 (3193.64)	7245.94 (3231.00)	7141.00 (3230.60)	7146.26 (3143.14)	7188.00 (3253.93)
Diastolic blood pressure (mm Hg), mean (SD)		77.99 (10.16)	77.20 (9.71)	78.14 (10.08)	76.98 (9.67)	78.55 (10.14)	77.11 (9.73)
Systolic blood pressure (mm Hg), mean (SD)		126.03 (14.10)	125.15 (13.30)	126.08 (13.83)	124.98 (13.31)	126.92 (13.65)	124.98 (13.65)

^a^Chi-square and *t* tests were computed to compare percentages and mean values from qualitative and quantitative variables, respectively, between the low and high categories for total sleep duration, deep sleep duration and deep/total ratio.

^b^All the corresponding *P* values were below .001 except for the heart rate variable where they were all above 0.05.

### Factors Associated With Poor Sleep

Age is strongly associated with sleep (see Table 2). Indeed, when compared to individuals aged between 18 and 30 years, the risk of poor total sleep was significantly increased up to the age of 60 years (OR 1.5, 95% CI 1.06-2.13) and tended to decrease for people age 60 years or more. Despite this, the linear trend is maintained (*P*=.024). On the other hand, age was not associated with a poor deep sleep duration except for the oldest category, where people aged 60 years or more had a lower risk of having a deep sleep less than 3 hours per night (OR 0.71, 95% CI 0.53-0.94). Finally, the risk of having a low sleep quality, evaluated by a deep/total sleep duration ratio below 0.40, decreased with age (*P*<.001). Individuals aged 60 years or more had a 53% reduction in the risk of having a low deep/total ratio (OR 0.47, 95% CI 0.35-0.63).

Women had a consistently lower risk of poor total sleep duration (OR 0.70, 95% CI 0.61-0.81), poor deep sleep (OR 0.51, 95% CI 0.45-0.58), and deep/total ratio (OR 0.45, 95% CI 0.38-0.54) compared to men. Weight was neither associated with deep sleep nor deep/total ratio. However, individuals with a weight over 98 kg, when compared to those under 75 kg, had a higher risk of having a poor total sleep duration (OR 1.17, 95% CI 1.03-1.33).

Aside from sex, heart rate was the most consistent factor associated with poor sleep. Indeed, high heart rate (>78 bpm) was associated with a poor total sleep quantity (OR 1.14, 95% CI 1.01-1.29), a poor deep sleep duration (OR 1.14, 95% CI 1.02-1.27), and a poor deep/total ratio (OR 1.18, 95% CI 1.04-1.34).

A high number of steps per day (>9028) and a high diastolic blood pressure (>83 mm Hg) were both associated exclusively with an increased risk of poor deep sleep (OR 1.13, 95% CI 1.01-1.27 and OR 1.21, 95% CI 1.06-1.39, respectively) when compared to a low number of steps and a low diastolic blood pressure but was not related to total sleep and deep/total ratio.

Systolic blood pressure was not related to total sleep or deep sleep but was associated positively with a poor deep/total ratio (*P*<.001). Individuals with a high systolic blood pressure (>133 mm Hg) had a 22% increased risk of having a deep/total sleep duration ratio below 0.40 (OR 1.22, 95% CI 1.04-1.43) when compared to those with a low one (≤116 mm Hg).

**Table 2:** Associations between cofactors and low total and deep sleep durations and deep/total ratio (N=15,839).

Factors		RIsk of low total sleep duration (≤6 hours)			Risk of low deep sleep duration (≤3 hours)			Risk of low deep/total ratio (≤0.40)		
		OR^a^	95% CI^b^	P value	OR	95% CI	P value	OR	95% CI	P value
**Age, years**				.024			<.001			<.001
	18-30	1	Ref		1	Ref		1	Ref	
	31-40	1.59	1.10-2.27		0.99	0.74-1.34		0.63	0.46-0.86	
	41-50	1.46	1.03-2.07		0.83	0.63-1.11		0.54	0.40-0.73	
	51-60	1.50	1.06-2.13		0.79	0.60-1.05		0.50	0.37-0.68	
	>60	1.33	0.94-1.89		0.71	0.53-0.94		0.47	0.35-0.63	
**Sex**										
	Male	1	Ref		1	Ref		1	Ref	
	Female	0.70	0.61-0.81		0.51	0.45-0.58		0.45	0.38-0.54	
**Weight (kg)**				.002			.162			.992
	≤75	1	Ref		1	Ref		1	Ref	
	76-86	0.94	0.84-1.07		0.96	0.86-1.07		0.96	0.84-1.09	
	87-98	0.98	0.86-1.11		0.97	0.87-1.09		0.92	0.80-1.05	
	>98	1.17	1.03-1.33		1.05	0.94-1.18		0.97	0.85-1.12	
**Heart rate (bpm)**				.003			.005			.016
	≤61	1	Ref		1	Ref		1	Ref	
	62-68	1.00	0.89-1.12		1.02	0.91-1.13		1.01	0.89-1.15	
	69-78	1.17	1.05-1.32		1.10	0.99-1.22		1.05	0.93-1.19	
	>78	1.14	1.01-1.29		1.14	1.02-1.27		1.18	1.04-1.34	
**Steps (n/day)**				.170			.081			,441
	≤4890	1	Ref		1	Ref		1	Ref	
	4891-6737	0.95	0.84-1.07		1.00	0.89-1.12		1.00	0.88-1.14	
	6738-9028	1.05	0.93-1.18		1.09	0.98-1.22		0.97	0.85-1.10	
	>9028	1.07	0.94-1.20		1.13	1.01-1.27		0.99	0.87-1.13	
**Diastolic blood pressure (mm Hg)**				.083			<.001			.005
	≤70	1	Ref		1	Ref		1	Ref	
	71-77	1.03	0.91-1.17		0.95	0.85-1.06		0.91	0.79-1.04	
	78-83	1.05	0.92-1.20		1.06	0.93-1.19		1.01	0.87-1.17	
	>83	1.10	0.95-1.28		1.21	1.06-1.39		1.12	0.95-1.32	
**Systolic blood pressure (mm Hg)**				.305			.222			<.001
	≤116	1	Ref		1	Ref		1	Ref	
	117-124	0.93	0.82-1.05		0.98	0.87-1.09		1.08	0.94-1.24	
	125-133	0.98	0.86-1.12		0.96	0.85-1.08		1.15	0.99-1.33	
	>133	0.99	0.85-1.14		1.00	0.88-1.15		1.22	1.04-1.43	

^a^OR: odds ratio.

^b^CI: confidence interval.

## Discussion

### Principal Findings

Based on data from more than 15,000 highly connected Withings customers, we were able to study factors associated with poor sleep, evaluated by the total sleep duration, the deep sleep duration, and the deep/total ratio as a proxy of sleep quality.

We approximated sleep quality with the deep/total sleep duration ratio. Those at risk of having a ratio value indicating poor sleep were males, younger individuals, and those with elevated heart rate and systolic blood pressure. It is usually reported that women have a poorer self-reported sleep quality when compared to men, especially when aged older than 50 years [30].

In accordance with our findings, heart rate and its variation, which are modulated by the combined effects of the sympathetic and parasympathetic nervous systems, have been previously associated with poor sleep [31]. In a previous report, blood pressure was not associated with sleep duration [29], which is consistent with our findings with total sleep duration, but we did find an association between diastolic blood pressure and poor deep sleep and another between systolic blood pressure and deep/total sleep duration ratio. It has been previously shown that women tended to report poorer quality of sleep than men [30,32]. Indeed, the poorer self-reported sleep quality in women appears to be partly mediated by their increased risk of depression and anxiety symptoms and in part to social factors such as socioeconomic status [33]. In comparison to self-report, women tend to have better sleep than men across a wide age range when sleep is evaluated with polysomnographic recordings, suggesting that objective and subjective assessments are tapping into different constructs of sleep. We are in agreement with the latest point. The decreased risk of poor sleep observed in women in our work raises the fact that passive information collected from connected devices can actually help to capture a more objective measure of sleep quality than the subjective measure collected by questionnaire. Therefore, these devices could serve as substitutes to polysomnography measurements at a much lower cost in large real-life observational or intervention studies.

### Strengths and Limitations

This study has some limitations. First, the study sample was mainly composed of men; a similar study should be reproduced in another population with a larger proportion of women. The analysis was also limited by the number of factors available to identify determinants associated with sleep. Some of these factors such as the sleep duration and number of steps can also be prone to biases in specific situations. Unfortunately, we did not have access to the algorithm for sleep evaluation or other determinants, so we were not able to retrieve information on how these variables were computed. But previous validation studies demonstrated that the correlation between total sleep duration evaluated by Withings devices and polysomnography, considered here as the gold standard in sleep evaluation, was strong (rho=0.94) [27]. Similarly, a high correlation between data on steps from the Withings Pulse activity tracker and an ActiGraph GT3X+ (rho=0.99) has been previously reported [34]. Nevertheless, our results confirm that the connected devices can be a useful tool to track sleep in large populations and identify sleep determinants and associated risk factors [35].

### Perspectives and Conclusion

We have been able to highlight several factors associated with either total or deep sleep duration and with the ratio deep/total sleep as a proxy of sleep quality. Because poor sleep is one of the lifestyle factors that is often neglected but in our modern society is associated with many chronic conditions, providing useful services to decrease its prevalence can be easily implemented through wearables devices. Even if these devices are imperfect, this study has shown as a proof of concept for further large population studies that tracking physical activity, anthropometry, and sleep with the help of mainstream connected devices is feasible and rich with information. We recommend other studies replicate our findings to have a consistent set of determinants of poor sleep measured from connected devices in different subpopulations.

Besides, these wearables could serve as prevention tools—for instance, with an alert or mobile phone notification system based on poor sleep risk factors. Such a service could be evaluated in future intervention studies to quantify its benefits in terms of improvement of sleep quality and quantity.

## Abbreviations

CI: confidence interval
OR: odds ratio
